# Ratiometric Electrochemical Sensor for Butralin Determination Using a Quinazoline-Engineered Prussian Blue Analogue

**DOI:** 10.3390/ma16031024

**Published:** 2023-01-23

**Authors:** Marcio Cristiano Monteiro, João Paulo Winiarski, Edson Roberto Santana, Bruno Szpoganicz, Iolanda Cruz Vieira

**Affiliations:** Department of Chemistry, Federal University of Santa Catarina, Florianópolis 88040-900, SC, Brazil

**Keywords:** Prussian blue analogue, quinazoline, ratiometric sensor, butralin

## Abstract

A ratiometric electrochemical sensor based on a carbon paste electrode modified with quinazoline-engineered ZnFe Prussian blue analogue (PBA-qnz) was developed for the determination of herbicide butralin. The PBA-qnz was synthesized by mixing an excess aqueous solution of zinc chloride with an aqueous solution of precursor sodium pentacyanido(quinazoline)ferrate. The PBA-qnz was characterized by spectroscopic and electrochemical techniques. The stable signal of PBA-qnz at +0.15 V vs. Ag/AgCl, referring to the reduction of iron ions, was used as an internal reference for the ratiometric sensor, which minimized deviations among multiple assays and improved the precision of the method. Furthermore, the PBA-qnz-based sensor provided higher current responses for butralin compared to the bare carbon paste electrode. The calibration plot for butralin was obtained by square wave voltammetry in the range of 0.5 to 30.0 µmol L^−1^, with a limit of detection of 0.17 µmol L^−1^. The ratiometric sensor showed excellent precision and accuracy and was applied to determine butralin in lettuce and potato samples.

## 1. Introduction

Prussian blue (PB) was the first polymeric coordination compound recorded in the literature by Diesbach and Dippel in the early 18th century [1]. The different oxidation states between iron atoms coordinated by a cyanide bridge give PB its characteristic blue color due to an intervalence transition around 720 nm [2]. A Prussian blue analogue (PBA) is the result of changes in the chemical composition of PB. When Fe^2+^ and/or Fe^3+^ ions are replaced by other different transition metal centers, such as cobalt, nickel, and zinc [3], it is also possible to change its properties by making small changes in its composition (and consequently in its structure): replacing the metallic centers and/or a CN^−^ group with other ligands, such as those of quinazoline [4].

Quinazoline (qnz, 1,3-diazanaphthalene) is a heterocyclic hybrid that has the molecular formula C_8_H_6_N_2_, and it is an important bicyclic skeleton structure in manifold natural products [5,6]. The quinazoline ring is formed by the union of a benzene ring with a six-membered ring containing 2 N atoms and contains three main isomers, namely, quinoxaline, cinnoline, and phthalazine [5]. Quinazoline and its derivatives have multiple biological activities and show a high affinity for metal ions; they also form all kinds of coordination compounds with sundry transition metals [7]. Regarding the application in electrochemical sensors, introducing nitrogen moieties into the electrode composition has obtained considerable interest, as it leads to the improvement of conductivity and of the electroactive area of the sensor, hence further boosting its electrochemical performance [8]. Therefore, quinazoline is an interesting ligand to be explored in the development of novel PBA for application in the field of electrochemical sensors.

Research on PBA composites and their derivatives has kept growing in the past decade, and they have been applied in energy conversion, energy storage, adsorption, and electrochemical sensors [9]. Regarding the application of electrochemical sensors, for PB/PBA, both oxidized and reduced forms have catalytic activity [3]. In addition, their zeolitic form has a channel diameter of approximately 3.2 Å and a cubic unit cell of 10.2 Å, allowing the diffusion of ions by the structure [3,9]. Furthermore, their high electronic transfer rate is another benefit, which is directly associated to the insertion/disinsertion of small ions [3]. Li et al. [10] developed a PBA-modified glassy carbon electrode for 2-nitrophenol determination. The synthesized PBA (K_x_Ni[Fe(CN)_6_]⋅nH_2_O) provided the electrochemical sensor with a higher electrocatalytic performance for the 2-nitrophenol reduction in comparison to bare GCE, which could be attributed to the better intrinsic catalytic nature of Ni, improved conductivity, and larger electroactive area.

Traditional electrochemical sensors usually depend on the precise measurement of a single current intensity, which further leads to low repeatability, reliability, and accuracy, and occasionally false negative results [11]. For this reason, ratiometric electrochemical sensors have recently attracted extensive attention [12,13,14,15]. These special sensors quantify the analyte with ratiometric a record of two signals (one is from the analyte and the other is from the inner reference). A peak intensity ratio (*I*_analyte_/*I*_inner reference_) is used as the measurement criteria for analytes [13]. Commonly, this ratiometric strategy reduces the intrinsic errors or background electric signals and exhibits a significant ability to further improve the accuracy and precision of the measurements [13,14]. Constant current responses from the internal reference can also indicate that the electrode surface remains homogeneous [14]. Consequently, ratiometric electrochemical sensors are considered more reliable and accurate than common electrochemical sensors [16]. PB has been used as an internal reference for ratiometric electrochemical sensors [15]. However, reports of works using these materials for ratiometric sensors are still limited.

In that regard, the detection of butralin (BTL) is of great importance, since BTL is a dinitroaniline herbicide applied in pre-emergence management of pests in manifold crops such as cotton, sunflower, rice, peanuts, corn, and vegetable crops [17,18]. Dinitroaniline herbicides are slightly soluble in water and moderately persistent in the environment by adsorbing to soil particles, such as organic matter, so it presents an environmental pollution and a potential threat to human health [19,20,21]. Regarding electrochemical sensors dedicated to the determination of BTL, only two works are found in the literature. Sreedhar and Reddy [22] developed a polarographic method for BTL determination using a dropping mercury electrode, and Gerent et al. [17] used a glassy carbon electrode modified with Co-Ag bimetallic nanoparticles stabilized in poly(vinylpyrrolidone). The electrochemical methods are greatly attractive because of their advantages, such as quick detection, convenient operation, cheap instrumentation, facile integration, and portability [23,24,25]. Therefore, the development of novel electrochemical tools to detect and supervise the dissipation behavior of BTL in edible raw food and in the environment is relevant.

In this work, the use of the quinazoline ligand and the metals Fe and Zn coordinated by the cyanide bridge was chosen to synthesize a novel PBA. To the best of our knowledge, this is the first report to the use quinazoline ligand to the synthesis of a PBA. Here, the PBA was incorporated into a carbon paste electrode and boosted the conductivity of the system, thus providing greater current intensities and, consequently, greater sensitivity and also serving as an internal reference to improve the precision and accuracy of the novel ratiometric sensor in the determination of BTL.

## 2. Materials and Methods

### 2.1. Reagents and Solutions

All reagents used in the experiment were analytical grade and purchased from commercial sources. Acetone, ethanol, sodium iodide, and sodium nitroprusside were purchased from Neon, Cambridge, MA, USA. Chloridric acid, zinc(II) chloride, iron(III) chloride, potassium chloride, sodium chloride, DMSO, butralin, and quinazoline were purchased from Merck, Darmstadt, Germany. The aqueous solutions were prepared with ultrapure water (18.2 MΩ cm), obtained with the Milli-Q system (Millipore, St. Louis, MO, USA). A stock solution of 10.0 mmol L^−1^ butralin was prepared in acetone and stored at 4 °C. Britton–Robinson (B–R) buffer (H_3_BO_3_, CH_3_COOH, H_3_PO_4_) (0.1 mol L^−1^) was used as the supporting electrolyte. The pH adjustments were performed with 6.0 mol L^−1^ HCl or NaOH.

To build the carbon paste electrode, Acheson 38 graphite powder (Fisher Scientific, Waltham, MA, USA) served as the conductor, and Nujol mineral oil (Merck, Darmstadt, Germany) served as a binding agent.

### 2.2. Synthesis and Characterization of PBA-qnz

The precursor complex pentacyanido(quinazoline)ferrate (PCF-qnz) was synthesized by solubilizing 0.58 mmol of PCF-amine in 1.0 mL of distilled water and mixing it with 1.0 mL of aqueous quinazoline solution (0.29 mmol). The reaction solution was kept under stirring, out of the reach of light, and in an ice bath for 30 min. After this period, 0.67 mmol of sodium iodide was added to the solution, and then 30 mL of ethanol was slowly added. The precipitated solid was filtered in a vacuum pump, washed with ethanol, and kept in a desiccator until a constant mass was obtained.

The Prussian blue analogue derivative from quinazoline ligand and zinc(II) (PBA-qnz) was synthesized by the direct method, which consists of mixing an excess aqueous solution of zinc chloride (0.40 mmol) with an aqueous solution of PCF-qnz (0.10 mmol) under agitation. After 15 min, the solid was precipitated with acetone and isolated by centrifugation.

The compounds were characterized by UV-Vis spectroscopy using a Lambda 35 spectrometer (Perkin Elmer, Waltham, MA, USA) with quartz cuvettes of 1.0 cm of optical length. FTIR was used to verify the main functional groups of both compounds, using a FTLA 2000 spectrophotometer (Asea Brown Boveri, Zürich, Switzerland). Electron paramagnetic resonance (EPR) spectra were obtained using an EMX micro-9.5/2.7 spectrometer (Bruker, Billerica, MA, USA) with a highly sensitive cylindrical cavity, operating in X-band (9 GHz), at 120 K, with 5 mW microwave power, 5 G modulation amplitude, and 100 kHz modulation frequency. Cyclic voltammetry and electrochemical impedance spectroscopy (EIS) measurements were performed in an Autolab PGSTAT128N potentiostat (Metrohm Autolab B.V., Utrecht, The Netherlands). EIS measurements were performed using the K_3_[Fe(CN)_6_]/K_4_[Fe(CN)_6_] redox probe (5.0 mmol L^−1^ equimolar mixture) in 0.1 mol L^−1^ KCl. For the EIS measure, the OCP was applied with a perturbation amplitude of 10 mV between the frequencies of 100,000 Hz and 0.1 Hz.

### 2.3. Construction of Electrochemical Sensor

Studies by our group have described the construction of sensors based on carbon paste [26]. The construction procedure for the sensor involved hand-mixing 18 mg of PBA-qnz (10% *w*/*w*) and 135 mg of graphite powder (75% *w*/*w*) for twenty minutes. After that, 27 mg (15% *w*/*v*) of Nujol was added and hand-mixed for 20 min more in a mortar. The resulting composite was packed firmly into the cavity of a syringe (3.0 mm inner diameter), and a copper wire was inserted to establish electrical contact. For comparison purposes, PCF-qnz/CPE and bare CPE were prepared using a similar procedure.

### 2.4. Electrochemical Measurements

The electrochemical measurements for the development of the analytical method for BTL were performed using a portable potentiostat PalmSens 4 (Palm Instruments BV, Houten, The Netherlands). The assays were carried out with a system of three electrodes: the proposed sensor (PBA-qnz/CPE) as the working electrode, a platinum plate as the auxiliary electrode, and Ag/AgCl (3.0 mol L^−1^ KCl) as the reference electrode. All assays were carried out at room temperature (25 ± 0.5 °C) in an electrochemical cell containing 10.0 mL of B–R buffer (0.1 mol L^−1^; pH from 2.0 to 7.0), and successive additions of a standard solution of BTL were carried out using a micropipette. Nitrogen gas was purged to the supporting electrolyte for 10 min before the assays.

### 2.5. Determination of BTL in Lettuce and Potato Samples

Fresh samples of lettuce (*Lactuca sativa*) and potato (*Solanum tuberosum*) were acquired from a farmers’ market in Florianópolis, Brazil. The lettuce and potato samples were prepared as follows: a mixture of 5.0 g of each vegetable with 25.0 mL of acetone was crushed in a blender for 5 min. The extract was filtered (25.0 μm) two times and diluted in acetone in a 50.0 mL volumetric flask for the analysis. For the assays, 500 µL of the samples was added to the electrochemical cell with 9.5 mL of 0.1 mol L^−1^ B–R buffer (pH 2.0).

## 3. Results and Discussion

### 3.1. Characterization of PCF-qnz and PBA-qnz

**Figure 1:**
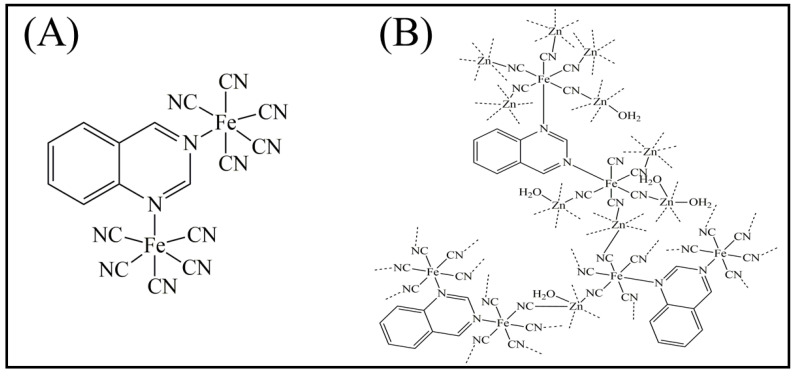
Structures of (**A**) pentacyanidoferrate with quinazoline ligand (PCF-qnz) and (**B**) Prussian blue analogue derivative from quinazoline ligand and zinc(II) (PBA-qnz).

**Figure 2:**
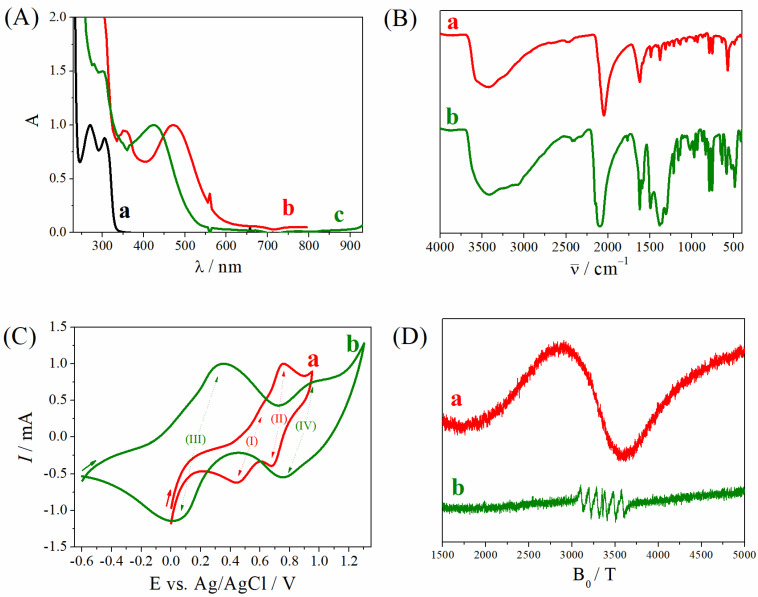
(**A**) UV-Vis spectra of (a) qnz, (b) PCF-qnz, and (c) PBA-qnz in aqueous solution. (**B**) Infrared absorption spectra of (a) PCF-qnz and (b) PBA-qnz. (**C**) Cyclic voltammograms of (a) PCF-qnz/CPE and (b) PBA-qnz/CPE with a scan rate of 25 mV s^−1^ (supporting electrolyte: 0.1 mol L^−1^ KCl). (**D**) EPR spectra in the X-band at room temperature of (a) PCF-qnz and (b) PBA-qnz, revealing the nature of the iron sites.

The PCF-qnz complex (Figure 1A) and PBA-qnz (Figure 1B) were first characterized using UV-Vis spectroscopy (Figure 2A). The PCF-qnz complex formed by the exchange of NH_3_ ligand for qnz ligand exhibits two bands of metal–ligand charge transfer in the visible region (355 and 474 nm, with log ε_max_ equal to 3.33 and 3.40, respectively). One of the characteristic bands of quinazoline [27] has a hypochromic shift when coordinating with the Fe atom, from 271 nm to 290 nm in PCF-qnz and to 280 nm in PBA-qnz.

Infrared spectra (Figure 2B) show that the PCF-qnz complex (curve a) exhibits characteristic bands of benzene (1378–1487 cm^−1^) and a pyrimidine ring (1580–1617 cm^−1^) [28]. Furthermore, it is possible to observe the CN^−^ (2047 cm^−1^) and Fe-CN (568 cm^−1^) stretches in the complex. Evaluating the FTIR data of PBA-qnz (curve b), it is possible to observe the presence of vibrations, referring to the vibrations of benzene at 1305–1492 cm^−1^ and the pyrimidine ring of qnz at 1592–1619 cm^−1^. The CN^−^ stretch can be observed at 2094 cm^−1^, as well as the Fe-CN-Zn stretch at 485 cm^−1^. Finally, the broadening of the ν(CN^−^) band in PBA-qnz means a variety of cyanides in the structure [29].

Cyclic voltammetry was used to study the electrochemical behaviors of PCF-qnz complex and PBA-qnz in 0.1 mol L^−1^ KCl (Figure 2C). Pentacyanidoferrates have a well-defined electrochemical process that is influenced by the nature of the ligand. The PCF-qnz complex (curve a) has a half-wave potential (E_½_) of 545 mV (I) and 720 mV (II) vs. Ag/AgCl, assigned to the pairs [Fe^2+/3+^(CN)_5_(qnz)Fe^2+/3+^(CN)_5_]^6−/4−^. The increase in potential represents a greater difficulty in removing electron density from iron due to the presence of the heterocyclic ligand [29]. Regarding the PBA-qnz (curve b), the E_½_ values were 180 mV (III) and 860 mV (IV) vs. Ag/AgCl. The fully reduced form of PBA-qnz, Zn[Fe^2+^(CN)_5_(qnz)Fe^2+^(CN)_5_] was oxidized at +183 mV vs. Ag/AgCl to form Zn[Fe^3+^(CN)_5_(qnz)Fe^2+^(CN)_5_]. Due to the presence of quinazoline in its structure, there is an increase in the amount of water coordinated, resulting in a lower σ-donor contribution, consequently resulting in a shift in the oxidation potential to more positive values, compared to traditional Prussian blue [30]. At +906 mV vs. Ag/AgCl, the second metallic center is oxidized, formatting Berlin green (Zn[Fe^3+^(CN)_5_(qnz)Fe^3+^(CN)_5_]). These processes occurred at more positive potential values than the traditional Prussian blue; in other words, the oxidation of PBA-qnz required a more positive potential value, indicating the coordination of the metal centers with the qnz ligand.

Although PCF compounds present iron atoms with the 2+ oxidation state, a broad signal around g ~2.021 can be observed for PCF-qnz. As it is a metal with six electrons, a value of g greater than g_e_ is expected [31]. This result occurs due to the magnetic interaction between iron ions, suggesting an Fe–Fe (spin–spin) interaction. The X-band EPR spectrum measured at room temperature reveals a profile similar to the EPR spectrum for PCF-amin (g ~2.2–2.3) presented by Ghobadi et al. [32]. The decrease in the value of g when exchanging the NH_3_ ligand for qnz suggests that binding with a compound that contributes to a strong field favors the MLCT process Fe^II^-qnz → Fe^III^-qnz. When analyzing the EPR spectrum of PBA-qnz (Figure 2D), seven peaks are observed. Zinc atoms fully occupy d orbitals, exhibiting no signs. Thus, the signs suggest a mixture of Fe^2+^ and Fe^3+^ valence states, with g values ranging from ~0.185 to ~0.214 [33]. These results agree with cyclic voltammetry.

### 3.2. Electrochemical Characteristics of PCF-qnz and PBA-qnz

**Figure 3:**
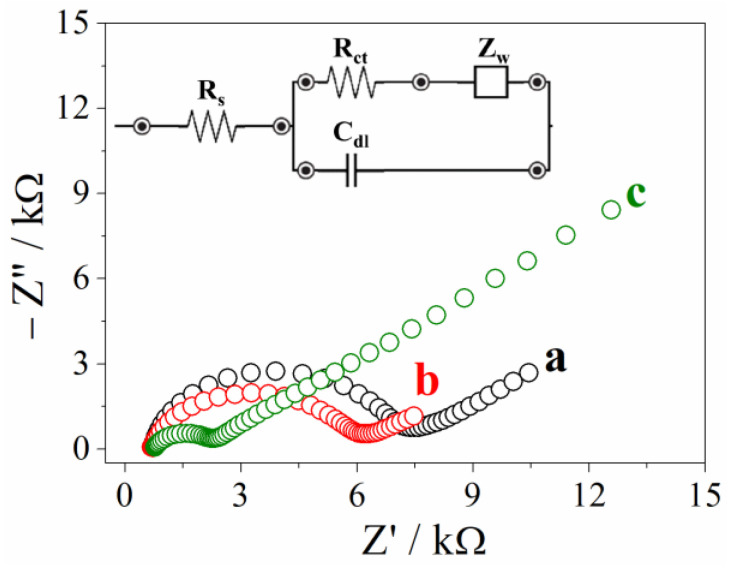
Nyquist plots for 5.0 mmol L^−1^ equimolar mixture of K_3_[Fe(CN)_6_]/K_4_[Fe(CN)_6_] in 0.1 mol L^−1^ KCl: (a) CPE, (b) PCF-qnz/CPE, and (c) PBA-qnz/CPE. We inserted the Randles circuit model for the electrodes. R_s_: solution resistance; R_ct_: charge-transfer resistance; Z_w_: Warburg impedance; C_dl_: double-layer capacitance.

EIS is a useful tool to investigate the interface properties of surface-modified electrodes. Nyquist plots were obtained in [Fe(CN)_6_]^3−/4−^ solution in KCl 0.1 mol L^−1^ for the following electrodes: (a) CPE, (b) PCF-qnz/CPE, and (c) PBA-qnz/CPE, which are shown in Figure 3. The charged transfer resistance (R_ct_) is positively correlated with the semicircle diameter in the high-frequency region of the EIS, and the diffusion process indicates the resistance offered by the mass transfer. Fitting the high-frequency region of the EIS plot, the R_ct_ of CPE is 6.1 kΩ (curve a). A reduced charge transfer resistance value of approximately 1000 Ω was observed for PCF-qnz/CPE (curve b), confirming the improved electrical conductivity based on the complex of Fe(II) and qnz. The R_ct_ for PBA-qnz/CPE is 1484 Ω (curve c), which is significantly lower than two working electrodes. This implies that, due to the polymerization of complex with Zn(II) moiety, the Prussian blue analogue becomes less resistive to charge transfer, increasing the electron transfer pathway between PBA-qnz/CPE and the redox probe. In addition, the introduction of nitrogen moieties into the electrode composition via quinazoline ligands leads to the improvement of conductivity, improving their electrochemical performance [8]. Thus, the PBA-qnz/CPE modified electrode can achieve an electrochemically sensitive determination of BTL.

### 3.3. Evaluation of Butralin Ratiometric Sensor Performance

**Figure 4:**
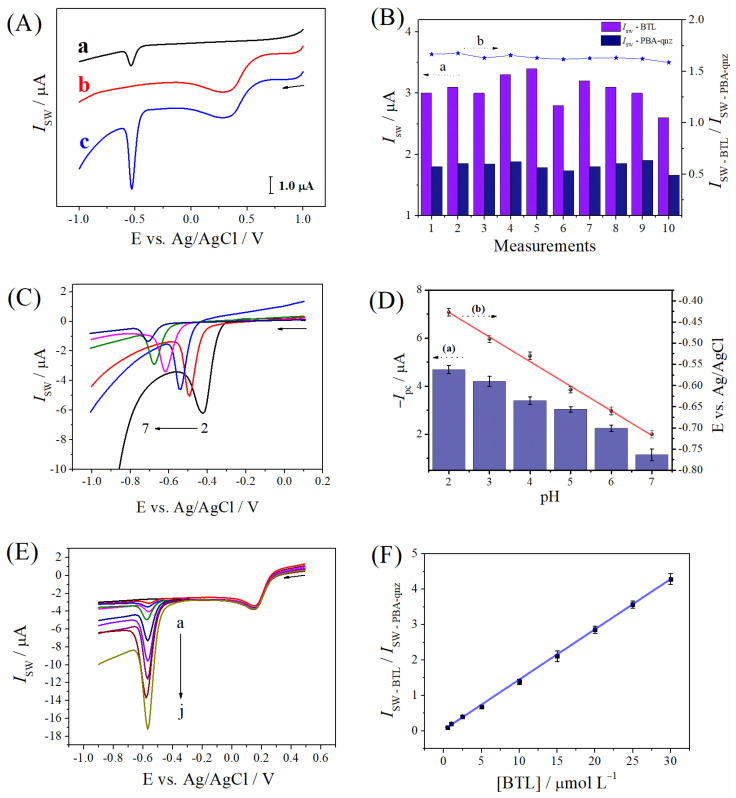
(**A**) Square wave voltammograms in the absence of BTL for (b) PBA-qnz/CPE in buffer solution and in the presence of 10.0 μmol L^−1^ BTL in B–R buffer (pH 2.0) for (a) CPE and (c) PBA-qnz/CPE. SWV parameters: *f* = 25.0 Hz, ΔEs = 1.0 mV, a = 50.0 mV (nonoptimized). (**B**) Current responses for *I*_SW − BTL_ and *I*_SW − PBA-qnz_ (axis a) and the *I*_SW − BTL_/*I*_SW − PBA-qnz_ ratio (axis b) using the same PBA-qnz/CPE on the same day. (**C**) Square wave voltammograms for 10.0 μmol L^−1^ BTL in B–R buffer using the PBA-qnz/CPE at different pH values. (**D**) Current (axis a) and potential (axis b) vs. pH (*n* = 3). (**E**) Square-wave voltammograms for BTL at PBA-qnz/CPE in 0.1 mol L^−1^ B–R buffer (pH 2.0): (a) blank, (b) 0.5, (c) 1.0, (d) 2.5, (e) 5.0, (f) 10.0, (g) 15.0, (h) 20.0, (i) 25.0, and (j) 30.0 μmol L^−1^ and (**F**) calibration plot (*n* = 3). SWV parameters: *f* = 50.0 Hz, ΔEs = 2.0 mV, a = 60.0 mV (optimized).

The electrochemical behavior of BTL was studied by square wave voltammetry (SWV) using the bare CPE and the PBA-qnz/CPE ratiometric sensor (Figure 4A). The square wave voltammogram exhibited a peak at −540 mV vs. Ag/AgCl, corresponding to the BTL reduction at bare CPE (curve a). The peak is correlated to the reduction of both nitro groups present in the molecule of BTL [17]. Using the PBA-qnz/CPE in the absence of BTL (curve b), a peak was recorded at +300 mV vs. Ag/AgCl, corresponding to the reduction of iron centers of the complex. Finally, when the BTL was analyzed using the PBA-qnz/CPE (curve c) a three-fold increase in the current intensities of the BTL compared to the performance of the CPE was recorded. This phenomenon can be attributed to the presence of nitrogen moieties in the electrode composition via quinazoline ligands, which leads to the improvement of conductivity and electroactive area of the sensor, boosting their electrochemical performance [8]. Even more important, PBA incorporated in the carbon paste was employed as a promising reference signal for the ratiometric sensor of BTL. The electroactive PBA can be oxidized to Berlin green or reduced to Prussian white at certain potentials and provide stable redox peaks, which can be used as an internal reference [15]. Thus, the measurements provided by the PBA-qnz/CPE ratiometric sensor show two signals (one is from the BTL analyte, and the other is from the PBA internal reference). The constant current responses of PBA-qnz inner reference indicated that the carbon paste was homogeneous, and consequently, the sensor surface was uniform, which contributes to better precision of measurements.

The repeatability of responses of the PBA-qnz/CPE sensor for BTL reduction was assessed across 10 consecutive measurements (Figure 4B). The plot shows a variation of current intensities for the BTL (axis a) with a relative standard deviation (RSD) of 8.0%. However, the ratio of *I*_SW − BTL_/*I*_SW − PBA-qnz_ (axis b) remained nearly unchanged (RSD = 1.5%), which indicates that the developed ratiometric sensing strategy minimized deviations among multiple assays because of the intrinsic built-in correction from the inner reference. This leads to a remarkable enhancement in the precision of the data provided by the sensor.

The effect of the pH of the supporting electrolyte on the electrochemical behavior of BTL was analyzed in the pH range of 2.0 to 7.0 (Figure 4C). Figure 4D (axis a) highlights how the cathodic peak currents vary as a function of the pH of the medium. It can be noted that the current values decreased from pH 2.0 to 7.0. Since hydrogen ions participate in the reduction of aromatic nitroanilines, the peak potential and intensities current of these compounds are pH-dependent. The pH of the medium influences the intensity and direction of the inductive and resonance effects operating in the molecule structure by changing the nature of the substituent [34]. The adsorption of nitroanilines onto the electrode surfaces in an acidic medium occurs because it has three different anchoring sites (the nitro, the amino function, and the aromatic ring system) [35]. Thus, it can be proposed that the adsorption of BTL onto the electrode surface occurs more efficiently at more acidic pH values, which results in higher current intensities. Another point to be considered is the lower stability of PBA-qnz as the pH of the medium increases, due to the affinity of OH^−^ ions for Fe(III) at pH close to 7.0, breaking the Fe^3+^−(CN)−Zn^2+^ bridge bond [36]. Thus, in aiming to obtain better sensitivity for the assays of BTL, pH 2.0 was selected for the subsequent analysis.

**Figure 5:**
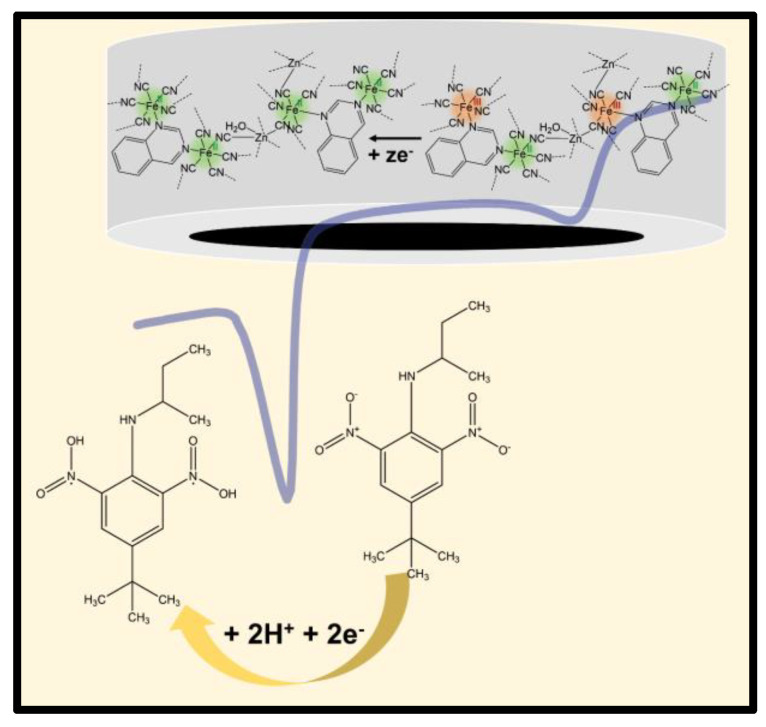
Proposed mechanism of BTL reduction on the surface of the PBA-qnz/CPE ratiometric sensor.

In addition, a linear shift of the E_p_ vs. pH plot (Figure 4C, axis b) was reached, with a slope of −58.1 mV pH^−1^, which was similar to the theoretical value of −59.2 mV pH^−1^ for the Nernst equation. These data indicate that an equal number of mols of electrons and protons are transferred during the reduction of BTL on the surface of the ratiometric sensor. According to the literature, both nitro groups of the molecule were simultaneously reduced, via the one-proton and one-electron mechanism for each nitro group [17]. A proposed reduction reaction for BTL is shown in Figure 5.

The calibration plot was built under optimized conditions at the PBA-qnz/CPE sensor after successive additions of the BTL standard solution (Figure 4E). The reduction peak of BTL can be observed at −0.57 ± 0.01 V vs. Ag/AgCl, with the current increasing proportionally to the species concentration, while the peak intensities referring to the reduction of the PBA-qnz remained stable at +0.15 V. The calibration plot was obtained in the range of 0.5 to 30.0 µmol L^−1^ (r = 0.998) (Figure 4F), and the equation for this plot can be expressed as *I*_SW − BTL_/*I*_SW − PBA-qnz_ = 0.05 (±2.0 × 10^−3^) [BTL]/μmol L^−1^ + 0.03 (±2.6 × 10^−3^). The limits of detection and quantification (LOD and LOQ) were calculated according to LOD = 3 × Sb/B and LOQ = 10 × Sb/B, where Sb is the standard deviation of intercept and B is the slope of the calibration plot [37]. The LOD and LOQ values obtained were 0.17 and 0.54 µmol L^−1^, respectively.

The analytical methods reported for the quantification of BTL are mostly chromatographic (Table 1). Only two works dedicated to the determination of BTL using electrochemical methods were found in the literature [17,22]. In that regard, the LOD obtained with the PBA-qnz/CPE is between the values obtained by other studies. The CPE can be easily prepared and modified, and it can be easily cleaned by manual sanding on a sheet of paper. Furthermore, the use of the PBA modifying agent served to increase the analyte current intensities and as an internal reference for the development of the ratiometric sensor. Thus, in addition to being sensitive, the proposed ratiometric sensor presented excellent precision data.

### 3.4. Interference and Stability Assays

The interference of organic compounds in the electroanalysis of BTL was studied under optimized conditions. The assays were carried out in 0.1 mol L^−1^ B–R buffer (pH 2.0) containing 10.0 μmol L^−1^ BTL (−0.56 V vs. Ag/AgCl) in the presence of 2-nitrophenol (−0.48 V), 3-nitrophenol (−0.45 V), 4-nitrophenol (−0.45 V), and parathion (−0.43 V), which were added at a concentration 10 times higher than that of the BTL. The reduction peak potential of these interferents did not coincide with the reduction peak potential of BTL, and, in addition, the decrease in the *I*_SW − BTL_/*I*_SW − PBA-qnz_ ratio in the presence of these interferents ranged from −0.5 to −1.5%. The results revealed that the proposed ratiometric sensor is highly selective for BTL quantification in the presence of organic compounds.

The stability of the ratiometric sensor was also inquired by measuring its response to 10.0 μmol L^−1^ BTL over 120 days. After this period, the PBA-qnz/CPE maintained a percentage of *I*_SW − BTL_ of 90% and 95% of *I*_SW − BTL_/*I*_SW − PBA-qnz_ in relation to its first response. These results indicated that the ratiometric sensor has excellent stability, evidencing its competence for the quantification of BTL.

### 3.5. Quantification of BTL in Lettuce and Potato Samples

The quantification of BTL by SWV in fresh samples was performed using the PBA-qnz/CPE ratiometric sensor (Table 2). Assays were carried out in triplicate using the standard addition procedure. The presence of BTL was not detected in any sample. Recovery values were obtained between 94 and 110%. These data confirm the accuracy of the data provided by the analytical method. Furthermore, the slopes of the standard addition plots were similar to those of the calibration plot (Figure 4F), which indicated that there were no influences from the matrix species of fresh samples.

## 4. Conclusions

A ratiometric sensor based on carbon paste modified with Prussian blue analogue derived from quinazoline ligand and zinc(II) was developed for the determination of BTL. This is the first device based on carbon paste dedicated to the electroanalysis of this herbicide. The use of the PBA modifying agent served to increase the BTL current intensities and as an internal reference for the development of the ratiometric sensor. The ratiometric sensor showed excellent precision and accuracy data and adequate selectivity for BTL. All of these capacities indicate the viability of the use of the PBA-qnz/CPE in the determination of BTL.

## Figures and Tables

**Table 1:** Comparison of the performance of different analytical methods in the quantification of BTL.

Analytical Method	Tools	Matrix	LOD/nmol L^−1^	Ref.
Chromatographic	HPLC-UV with SPME ^a^	Surface water	0.2	[38]
Chromatographic	HPLC-UV-ESI/MS ^b^	Tobacco leaf powder	508	[39]
Immunochromatographic	Gold-based strip sensor	Phosphate buffer saline (pH 7.4)	10.4	[20]
Electrochemical	Co-Ag BMNPs-PVP/GCE ^c^	B–R buffer (pH 2.0)	32.0	[17]
Electrochemical	Dropping mercury electrode	B–R buffer (pH 4.0)	60.0	[22]
Electrochemical	PBA-qnz/CPE	B–R buffer (pH 2.0)	170	This study

^a^ HPLC-UV with SPME—High-performance liquid chromatography with ultraviolet detection with solid phase microextraction fiber coating based on silicone sealant/hollow ZnO@CeO_2_ composite; ^b^ HPLC-UV-ESI/MS—High-performance liquid chromatography with ultraviolet detection and electrospray ionization mass spectrometry; ^c^ Co-Ag BMNPs-PVP/GCE—Glassy carbon electrode modified with Co and Ag bimetallic nanoparticles immobilized in poly(vinylpyrrolidone).

**Table 2:** Determination of the level of BTL in fresh samples using the ratiometric sensor.

Samples	Determined ^a^ /µmol L^−1^	Added/µmol L^−1^	Found ^a^/µmol L^−1^	Recovery ^b^/%
Lettuce (*Lactuca sativa*)	Not detected	1.0	1.03	95–110
		10.0	10.07	99–102
Potato (*Solanum tuberosum*)	Not detected	1.0	1.01	94–110
		10.0	9.97	97–105

^a^ Mean of three measurements under the same conditions by SWV using the PBA-qnz/CPE; ^b^ Recovery = (amount found − amount determined)/amount added × 100.

## Data Availability

Not applicable.
